# Low-energy ionizing radiation and carcinogenesis: a call for optimized clinical stewardship in the computed tomography era

**DOI:** 10.3332/ecancer.2026.2103

**Published:** 2026-03-31

**Authors:** Satyajeet Ratha, Samarth Ozab, Sweta Sonic, Sneha Dhillon

**Affiliations:** Department of Radiation Oncology, All India Institute of Medical Sciences, Rajkot 360060, India; a https://orcid.org/0000-0003-3905-4922; b https://orcid.org/0009-0009-5727-2692; c https://orcid.org/0000-00001-8349-1957

**Keywords:** cancer epidemiology, computed tomography, radiation protection

## Abstract

Computed tomography (CT) scans are widely used diagnostics. Despite their diagnostic advantages, their increasing reliance necessitates critical examination of projected lifetime cancer risks due to radiation exposure, a stochastic effect linearly correlated with dose. CT-associated cancers are estimated to increase significantly. The paediatric population is disproportionately vulnerable, facing higher risks even from lower doses. Current risk models may underestimate cancer incidence due to Dose and Dose Rate Effectiveness Factor complexities. High-risk scans such as whole-body CT, abdominal CT and brain CT, significantly increase exposure. A critical re-evaluation of guidelines to prioritise the risk-benefit ratio for CT use. Future diagnostic and screening strategies must prioritize innovation in alternative non-ionizing modalities like magnetic resonance imaging, ultrasonography and thermography with integration of Artificial Intelligence. ‘As Low As Reasonably Achievable’ principle is essential, aiming to limit ionizing radiation doses to near zero to safeguard public health.

First clinical computed tomography (CT) Scan was used in 1971, and since then it has became a popular diagnostic tool due to its superiority not only in sensitivity and specificity but also cost effectiveness, lesser scan time and universal availability [1, 2]. Compared to its peers, e.g., ultrasonography (USG) and magnetic resonance imaging (MRI), which were also introduced in clinical use in 1970s and 1980s, respectively, CT Scan is admired due to its universal diagnostic capabilities and financial suitability. An estimated 93 million CT scans, nearly 40 million MRIs, and more than 100 million ultrasonography examinations were performed annually in the USA as per 2023 data. [3, 4]. The stochastic effect of radiation is a well-established principle, and it demonstrates a linear correlation between radiation doses and increased cancer risk [5]. Equation for cancer risk:

R _cancer_ = Effective dose (Sv) × Risk factor (5.5 × 10^−2^) (F) (ICRP)

Radiation exposure increases the risk of cardiovascular diseases and benign tumours, although current data are inadequate for risk quantification (BEIR VII) [6].

The increasing reliance on CT imaging, while a diagnostic triumph, necessitates a critical examination of its projected lifetime cancer risks. It is estimated that over 100,000 new cancer cases in the United States each year may be attributable to exposure from CT imaging, representing approximately 5% of all newly diagnosed cancers [4]. Notably, the paediatric population, especially those under 1 year of age, faces a disproportionately higher projected cancer risk, even from lower radiation exposure [6]. An Australian epidemiological study on paediatric population (0–19 age) estimated approximately 24% increased cancer incidence per CT scan [7]. This heightened vulnerability in children is attributed to their longer life expectancy, allowing more time for radiation-induced cancers to manifest and their greater cellular radio sensitivity [8]. A retrospective hospital study on surgical patients revealed that roughly 50% of CT scans were unremarkable [9], highlighting potential overuse and unnecessary radiation exposure.

The biological effects of ionizing radiation were mainly studied on The Japanese atomic bomb survivors and scientific committee BEIR VII reported that radiation induced carcinogenesis follows ‘Linear-No-Threshold’ (LNT) risk model meaning there will be ‘some’ risk even at lower doses of exposure. BEIR VII reported life time cancer risk of one in 100 individuals for single exposure of 100 mSv. This risk decreased proportionately as one in 1,000 individuals for single exposure of 10 mSv [10]. Current reports estimate lifetime cancer risk from CT scan based on standard BEIR VII and Radiation Risk Assessment Tool (RadRAT). RadRAT calculator was developed by National Cancer Institute which provides lifetime cancer risk and organ-specific risk estimates. As radiosensitivity and repair capacity of various organs are different, similar low dose exposure may exhibit different effects in organs. Schneider et al [11] proposed organ equivalent dose as LNT model does not effectively estimate cancer risk of specific organs except colon, cervix and skin. They reported excess relative risk of 1, 0.85 and 0.48 events per Gray (Gy) for female breast, colon and lung, respectively, for exposure at age of 30 and attained age of 70 years [11]. On a practical note, one individual may be exposed to different types of low-energy modalities (X Rays, Fluoroscopy, CT, Positron Emission Tomography and so on) at different intervals and to different body parts. Cumulative exposure doses may vary depending on individual bases. Ionizing Radiation Reporting System should be established, which collects site specific and organ-specific data for individuals and from all low-energy ionizing radiation types. This will generate more accurate data for more precise estimation. Sodickson et al [12] studied on cumulative CT scan doses in which 15% patients received ≥100 mSv and 4% patients received 250–1,375 mSv of cumulative exposure. A study showed additional cancer risk of 1 per 1,000 patients for 10 mSv exposure at age of 30 for females and 20 for males. For females below the age of 30 and males below the age of 20 excess cancer risk follows exponential trajectory with the highest risk reaching upto ~4.5 per 1,000 females and ~2.5 per 1,000 males [12]. Similarly, repeat CT scans done at intervals of 6–12 weeks can result in elevated levels of DNA repair foci which can elevate risk of malignancy [13]. In addition to diagnostic scans, imaging done to verify setup during radiotherapy treatment delivery also adds to the cumulative lifetime radiation dose. Specifically in Image Guided Radiation Therapy (IGRT) includes low energy cone beam CT (kvCBCT) scans for accurate matching and dose delivery. Kim et al [14] reported association of kvCBCT imaging doses and secondary cancer risk. They found lifetime attributed risk for in-field organs is in range of ~100–400 per 10,000 and for out-of-field organs it is ~2–4 per 10,000 for the pelvic region site (average in-field dose:16.7 cGy, average out-of-field dose: 0.67–0.02cGy) [14]. It shows IGRT imaging optimization is very important, specifically for paediatric age group.

Dose and Dose Rate Effectiveness Factor (DDREF) depends on two variables: Low Dose Effective Factor and Dose rate Effective Factor. Where low dose is defined as less than 100 mGy and low dose rate is defined as less than 1 mGy/min averaged over 1 hour. ICRP in 2007 accepted DDREF 2 as standard but various international bodies were not in agreement. BEIR VII and NAS in 2006 considered DDREF value as 1.5. Whereas WHO in 2013 reported use DDREF value 1 as standard. UNSCEAR was against the use of DDREF for calculation [15]. Impact on risk estimates is huge when DDREF values changes as estimates doubles when factor value one is used instead of two. Observed DDREF in nuclear worker epidemiological study done by Hoel *et al* [16] suggest DDREF 2.63. Nonlinear dose response curves at lower dose along with bystander effects makes it more complicated to understand effects of radiation at cellular level which may continue for many years or even decades [17]. Integration of epidemiological & radiobiological research is need of hour for stronger evidence [17]. Lower doses of radiation may cause non-cancerous effects such as inflammatory responses, which may be beneficial or harmful [18]. Recent advances in clinical radiation oncology e.g. FLASH radiotherapy also highlights very high dose rates effects are not comparable to routine conventional dose rate effects. To generate stronger and specific evidence epidemiological studies are essential.

With increasing life expectancy due to advancement in healthcare, use of diagnostic imaging is set to rise further. We must critically re-evaluate the risk-benefit ratio for CT use, particularly in paediatric age and for whole-body, abdominal and brain CT scans in adult age, given their association with higher cancer risk (Table 1).

Adherence to the ‘As Low As Reasonably Achievable’ principle for radiation protection is paramount. Screening CT adds considerable numbers of scans; therefore, the use of alternative screening tools should be encouraged. With 42 million screening mammograms performed last year in the USA alone [19], there is a compelling concern that this widespread screening contributes to the rising global incidence of breast cancer observed since its introduction [20]. Future strategies must prioritize innovation in alternative screening and diagnostic techniques – such as MRI, USG and thermography (for breast) – integrating Artificial Intelligence/Machine Learning to achieve comparable sensitivity, specificity, cost-effectiveness and feasibility to CT.

Our ultimate goal should be to limit ionising radiation doses to as close to zero as possible, safeguarding public health in the long term.

The following CT Usage Recommendations may be followed to minimize exposure:

- Prefer alternative non-ionising imaging modalities, particularly for the paediatric population
- Consideration of cumulative dose accumulation factor before prescribing scan – that requires patient-based unique data for the previous scan and exposure details
- In cases where CT imaging cannot be avoided, radiation dose optimization should be achieved by utilizing the latest hardware improvements and AI/ML-based software techniques.
- Hospital-based monthly and annual review of number of scans and reason for scan, particularly for paediatric age group.

## Conflicts of interest

The authors declares no conflicts of interest.

## Funding

No funding was obtained or allocated for the conduct of this study.

## Figures and Tables

**Table 1.** Ionizing radiation and approximate effective radiation dose [21].

Source of ionizing radiation	Approximate effective radiation dose
Background radiation (BR)	3 mSv (~0.008 mSv/day)
Dental X ray	0.005 mSv (1 day of BR)
Chest X ray	0.1 mSv ( 10 days of BR )
Screening mammography	~0.30 mSv (35–40 days of BR)
CT lung screening	1.5 mSv (6 months of BR)
CT chest	6.1 mSv (2 years of BR)
CT brain	1.6 mSv (7 months of BR)
CT spine	8.8 mSv (3 years of BR)
CT abdomen pelvis	7.7 mSv (2.6 years of BR)
PET CT scan	22.7 mSv (7.6 years of BR)
